# Altered Regional Cortical Brain Activity in Healthy Subjects After Sleep Deprivation: A Functional Magnetic Resonance Imaging Study

**DOI:** 10.3389/fneur.2018.00588

**Published:** 2018-08-02

**Authors:** Lingling Chen, Xueliang Qi, Jiyong Zheng

**Affiliations:** ^1^Department of Pediatric Internal Medicine, Linyi Central Hospital, Yishui, China; ^2^Department of Medical Imaging, The Affiliated Huaian No. 1 People's Hospital of Nanjing Medical University, Huaian, China

**Keywords:** sleep deprivation, receiver operating characteristic, area under the curve, amplitude of low frequency fluctuations, functional magnetic resonance imaging

## Abstract

**Objective:** To investigate acute sleep deprivation (SD)-related regional brain activity changes and their relationships with behavioral performances.

**Methods:** Twenty-two female subjects underwent an MRI scan and an attention network test at rested wakefulness (RW) status and after 24 h SD. The amplitude of low-frequency fluctuations (ALFF) was used to investigate SD-related regional brain activity changes. We used the receiver operating characteristic (ROC) curve to evaluate the ability of the ALFF differences in regional brain areas to distinguish the SD status from the RW status. We used Pearson correlations to evaluate the relationships between the ALFF differences in brain areas and the behavioral performances during the SD status.

**Results:** Subjects at the SD status exhibited a lower accuracy rate and a longer reaction time relative to the RW status. Compared with RW, SD showed significant lower ALFF values in the right cerebellum anterior lobe, and higher ALFF areas in the bilateral inferior occipital gyrus, left thalamus, left insula, and bilateral postcentral gyrus. The area under the curve values of the specific ALFF differences in brain areas were (mean ± std, 0.851 ± 0.045; 0.805–0.93). Further, the ROC curve analysis demonstrated that the ALFF differences in those regional brain areas alone discriminated the SD status from the RW status with high degrees of sensitivities (82.16 ± 7.61%; 75–93.8%) and specificities (81.23 ± 11.39%; 62.5–93.7%). The accuracy rate showed negative correlations with the left inferior occipital gyrus, left thalamus, and left postcentral gyrus, and showed a positive correlation with the right cerebellum.

**Conclusions:** The ALFF analysis is a potential indicator for detecting the excitation–inhibition imbalance of regional cortical activations disturbed by acute SD with high performances.

## Introduction

Sleep is a necessary physical need for normal life, and we spend nearly one-third of our life sleeping. Sleep deprivation (SD), widespread in the current society, is caused by environmental factors or personal reasons and generally has deleterious effects on emotional regulation, memory, attention, and executive control function (1–5). Long-term SD can lead to multiorgan and multisystem dysfunction and has been shown to have negative impacts on metabolic, physiological, psychological, and/or behavioral reactivity with a greater risk of being a serious disease (6–10). However, their mechanisms are still unclear.

Resting state functional MRI (rfMRI) does not need the use of radioactive tracers and can combine functional and structural images, making the imaging method suitable for exploring the mechanisms of and obtaining insights into the pathophysiology of diseases (3); furthermore, rfMRI can be used to find the location of altered neuronal spontaneous brain activity. Recently, numerous scholars have focused their attentions on whether short-term SD has detrimental effects on regional neuronal spontaneous brain activity and cognitive function. RfMRI studies have consistently found altered cognitive domains, and altered regional spontaneous brain activity and functional connectivity patterns in the sleep-deprived brain (3, 6, 11–19), suggesting that the internal brain activity and intra-/inter- connectivity patterns for the internal processing of information are disturbed by SD. Furthermore, recent studies have found that SD has accumulative negative effects on brain morphology and advanced cognitive function (attention and working memory), showing that as SD hours prolonged, more areas show reduced gray matter volume, and after one night's sleep the brain atrophy is restored and replaced by increased gray matter volume (10). However, few studies have considered the gender factor in the neuroimaging studies of sleep disorders, and both female and male subjects were combined in these studies. Thus, the neurological mechanism of the location of altered neuronal spontaneous brain activity based on gender has not been fully studied.

Amplitude of low-frequency fluctuations (ALFF) measurement has the ability to locate where (in which brain region) regional spontaneous brain activity was disturbed with less computation complexity and high test–retest reliability characterization (20–24). Theses characterizations may make the ALFF analysis a useful tool and potential indicator for rs-fMRI data to explore the various potential neurobiological mechanisms by locating the altered regional spontaneous brain activity and functional connectivity patterns (3). Recently, ALFF analysis has been successfully applied to the exploration of neural mechanism of primary insomnia (24), wakefulness and light sleep (25), and obstructive sleep apnea (22). In this framework, in the present study we hypothesized that the ALFF measurement has the ability to locate acute SD-induced regional brain activity with high sensitivity and specificity. To test this hypothesis, we used the ALFF analysis as a potential indicator to locate the underlying altered regional functional brain activity during the SD status relative to the rested wakefulness (RW) status, and further explored the potential neurobiological mechanisms of SD in female subjects with respect to the location of altered neuronal spontaneous brain activity. Specifically, the receiver operating characteristic (ROC) curve was used to investigate the abilities of the ALFF analysis in distinguishing the SD status from the RW status. Pearson correlations were used to evaluate the relationships between the ALFF differences in brain areas and the behavioral performances during the SD status.

## Materials and methods

### Subjects

The present study was approved by the Medical Research Ethical Committee. The Affiliated Huaian No.1 People's Hospital of Nanjing Medical University. Twenty-two healthy female subjects (age, 26.91 ± 6.05 years; education, 15.77 ± 1.15 years; mean ± std) were recruited. All subjects met the following criteria as in previous studies (3, 6): (1) right-handed; (2) good sleep habit without any symptoms of sleep disorders such as difficulties in initiating and/or maintaining sleep, with Pittsburgh sleep quality index score < 5; (3) never taken alcohol, stimulants, cigarette, hypnotic or psychoactive medications, diet pills, and caffeine for ≥3 months during and prior to the current study; (4) regular dietary habit with moderate weight and body shape; (5) without foreign implants, and inborn and acquired diseases.

Each of the subjects underwent the MRI scan twice, once during RW status and the other after 24 h acute SD. The acute SD session started from 19:00 p.m. on the first day and lasted until 7:00 p.m. on the second day. Before the MRI scan, all volunteers underwent an attention network test (26, 27). Food and water were provided during the SD procedure. The temperature of the room was maintained between 23 and 27°C. The staff of our team used video monitors and worked in turns to make sure that the participants did not fall asleep. If the participants showed signs of falling asleep, they were immediately awakened using an alarm clock by staff. A simple questionnaire was used to evaluate whether the subjects were asleep during the MRI scan. All subjects provided their written informed consent voluntarily.

### MRI

The MRI examination was performed using an acquired clinical 3.0-Tesla MRI scanner (SIEMENS Trio Tim, Siemens Healthcare, Erlangen, Germany) with a standard eight-channel head coil. First, we acquired a high-resolution 3D anatomical image with 176 T1-weighted images in a sagittal orientation: repetition time = 1,950 ms, gap = 0 mm, echo time = 2.3 ms, thickness = 1 mm, acquisition matrix = 248 × 256, flip angle = 9°, field of view = 244 × 252 mm. Second, we also acquired 240 functional images using a single-shot gradient-recalled echo-planar imaging pulse sequence (repetition time = 3,000 ms, gap = 0.5 mm, echo time = 25 ms, thickness = 5.0 mm, flip angle = 90°, acquisition matrix = 32 × 32, field of view = 210 × 210 mm).

### Data analysis

The first 10 time points of the functional images were discarded because of the possible instability of the initial MRI signal and to allow the participants to adapt to the scanning environment. Data preprocessing of the remaining resting-state images was performed using the Data Processing & Analysis for Brain Imaging (DPABI 2.1, http://rfmri.org/DPABI) toolbox, adopting the Digital Imaging and Communications in Medicine (DICOM) standard for form transformation, slice timing, head motion correction, spatial normalization, and spatial smoothing using a Gaussian kernel of 8 × 8 × 8 mm^3^ full-width at half-maximum. Participants with more than 1.5 mm maximum translation in x, y, or z directions and 1.5° of motion rotation were removed. After the head motion correction, the rest of the functional images were spatially normalized and resampled to Montreal Neurological Institute (MNI) space at a resolution of 3 × 3 × 3 mm^3^. Linear regression was applied to remove several sources of possible spurious covariates, including 24 head motion parameters obtained in the realigning step, signal from a region in the cerebrospinal fluid or/and centered in the white matter, and global signal averaged over the whole brain. After preprocessing, the time series were further linearly detrended and temporally band-pass filtered (0.01–0.1 Hz). The details of the ALFF calculation have been reported in previous studies (3, 28). To reduce the global effects of variability across the participants, the mean ALFF value of each voxel was divided by the global mean ALFF value for each participant.

### Statistical analysis

Data are presented as mean ± standard deviation (mean ± std). Pair *t*-tests were used for demographic factors (age, years of education, and clinical factors), and a chi-squared (χ^2^) test was used for categorical data (gender). *p* < 0.05 was considered to be a significant difference.

A pair *t*-test was used to investigate the ALFF differences in regional brain areas of the subjects during the acute SD status relative to the RW status with the gender, age, and years of education as nuisance covariates of no interest. AlphaSim correction (threshold of individual voxel of *p* < 0.01 and cluster level of *p* < 0.05 with contiguous voxel size ≥20) was used to determine the statistical differences.

We used the ROC curve to evaluate the ability of the ALFF differences in regional brain areas to distinguish the SD status from the RW status, and we used Pearson correlations to evaluate the relationships between the ALFF differences in brain areas and the behavioral performances during the SD status. The statistical threshold was set at *p* < 0.05.

## Results

### Behavioral characteristics

Compared with the RW status, the acute SD status had a lower response in accuracy rate (mean ± std, 24 h SD = 96.07 ± 3.2%, RW = 97.85 ± 1.69%; *t* = −2.125, *p* = 0.046) and a longer response in reaction time (24 h SD = 633.99 ± 79.05 ms; RW = 537.97 ± 46.49 ms; *t* = 5.554, *p* < 0.001).

### ALFF differences between groups

**Figure 1:**
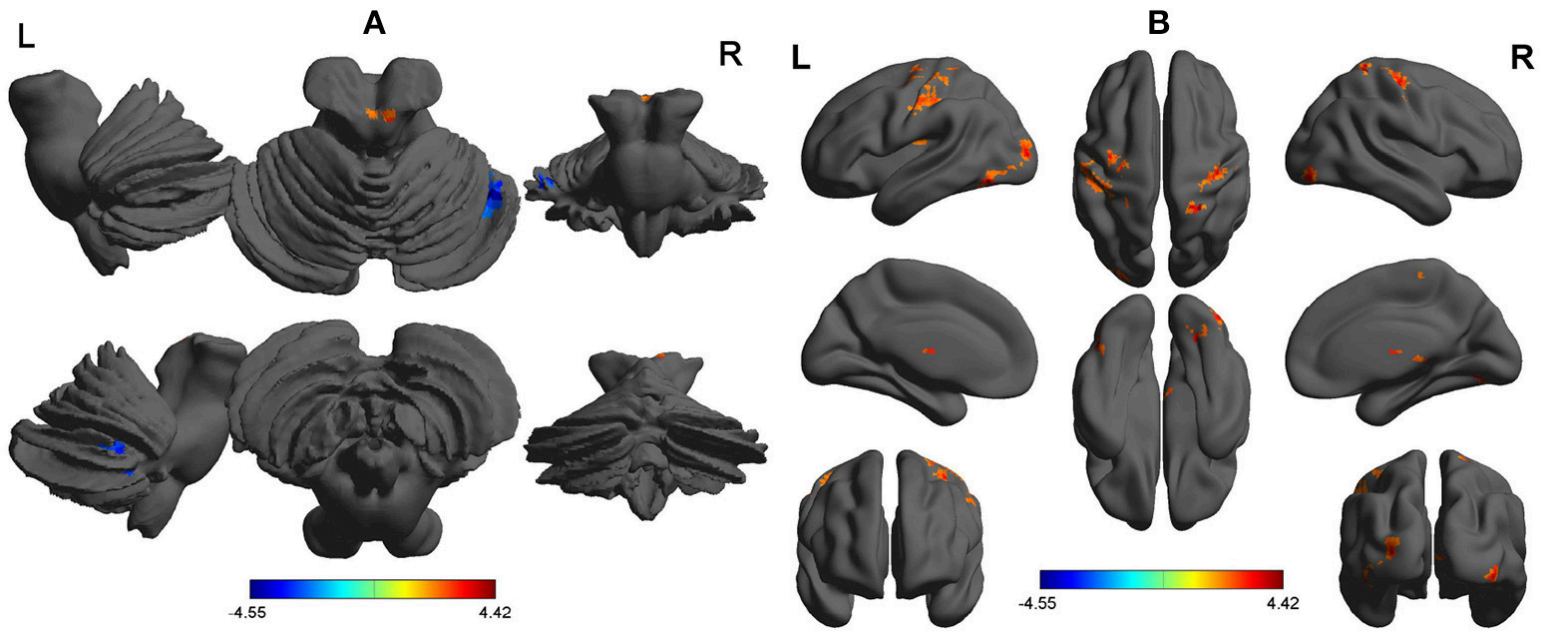
ALFF differences between SD group and RW group. The differences covered cerebellum with lower ALFF **(A)**, and inferior occipital gyrus, thalamus, insula, and postcentral gyrus with higher ALFF **(B)**. The color in the map represents the differences. The red color signifies increase in ALFF areas, and the blue signifies decrease in ALFF areas. ALFF, Amplitude of low-frequency fluctuations; SD, Sleep deprivation; RW, Rested wakefulness; R, right; L, left.

Compared with RW, SD had significant lower ALFF areas in the right cerebellum anterior lobe (Figure 1A), and higher ALFF areas in the bilateral inferior occipital gyrus (Brodmann's area, BA 18, 19), left thalamus, left insula (BA 13), and bilateral postcentral gyrus (BA 3, 6) (Table 1, Figure 1B).

**Table 1:** ALFF differences in brain areas between SD and RW.

**Brain regions of peak coordinates**	**R/L**	**BA**	**Voxel size**	***t*-score of peak voxel**	**Peak MNI coordinates**
					**X, Y, Z**
Cerebellum Anterior Lobe	R	N/A	33	−4.5537	39, −54, −33
Inferior Occipital Gyrus	L	18, 19	142	4.4223	−48, −84, −6
Inferior Occipital Gyrus	R	18, 19	74	3.5394	39, −84, −9
Thalamus	L	N/A	89	4.1044	−6, −24, 18
Insula	L	13	43	3.9142	−36, −18, 21
Postcentral Gyrus	L	3, 6	235	3.9225	−36, −27, 42
Postcentral Gyrus	R	3	230	4.2278	27, −45, 66

*The statistical threshold was set at corrected significance level of individual voxel p < 0.01 using an AlphaSim-corrected threshold of cluster p < 0.05*.

*ALFF, Amplitude of low-frequency fluctuation; SD, Sleep deprivation; RW, Rested wakefulness; R, right; L, left; BA, Brodmann's area; MNI, Montreal neurological institute; N/A, Not applicable*.

### ROC curve

**Figure 2:**
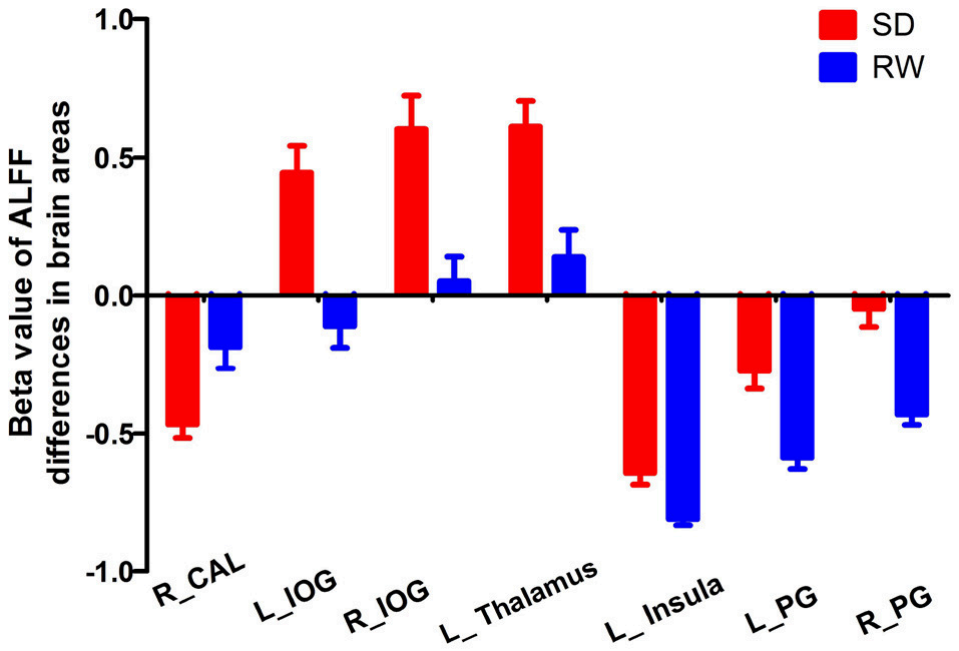
Mean beta values of ALFF differences in regional brain areas. Significant differences were found for beta value of all ALFF brain areas between SD and RW (*p* < 0.001). ALFF, Amplitude of low-frequency fluctuations; SD, Sleep deprivation; RW, Rested wakefulness; R, right; L, left; CAL, Cerebellum anterior lobe; IOG, Inferior occipital gyrus; PG, Precentral gyrus.

The mean beta value of ALFF differences in the altered areas were extracted (Figure 2). These different ALFF differences in brain areas were further used for the ROC curve to evaluate their abilities to distinguish the acute SD status from the RW status. The area under the curve (AUC) values of those specific ALFF differences in brain areas were (0.851 ± 0.045; 0.805–0.93).

**Figure 3:**
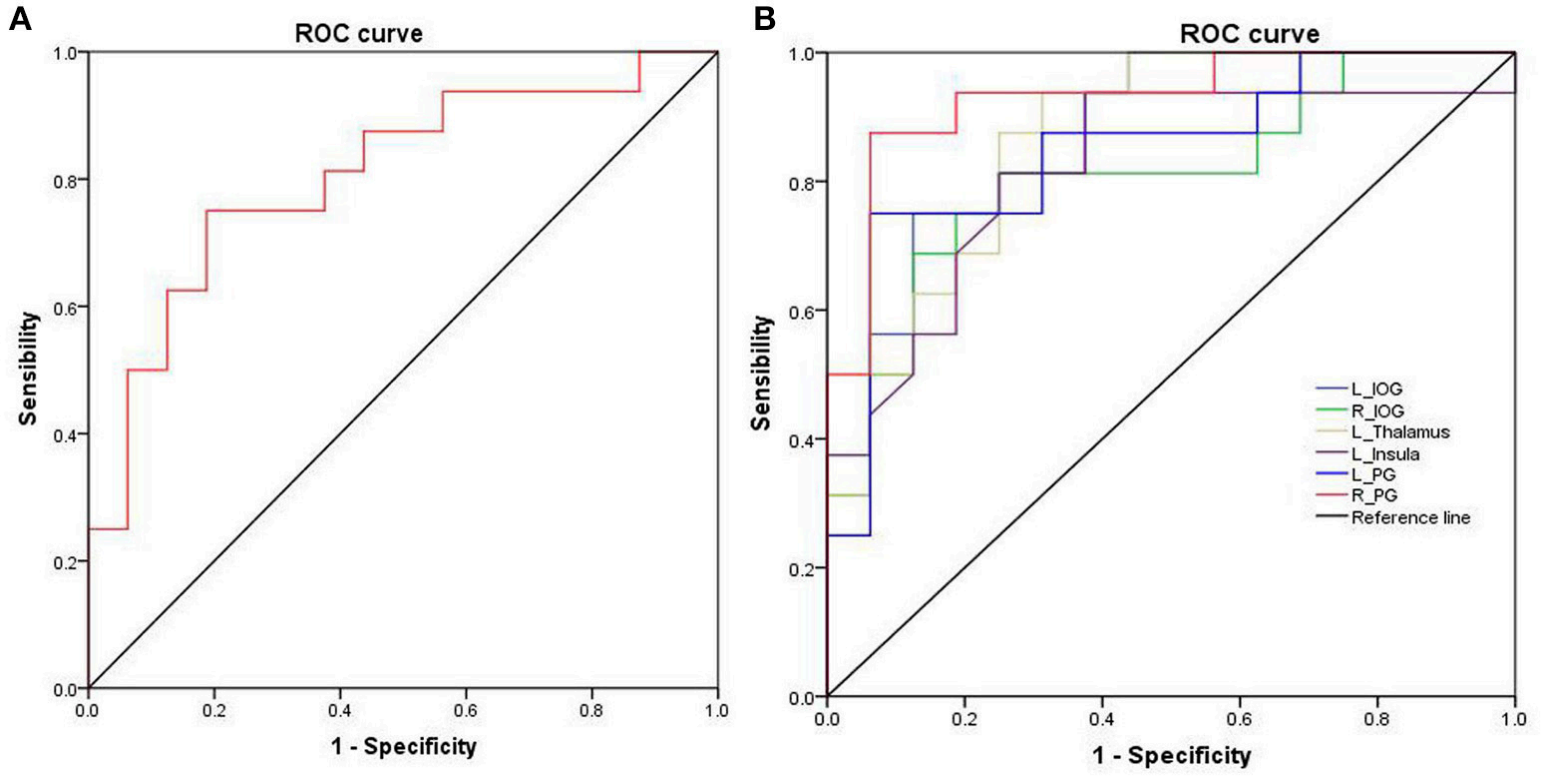
ROC curve analysis of ALFF differences in regional brain areas. ROC curve of cerebullum **(A)** and other areas **(B)**. ROC, Receiver operating characteristic; ALFF, Amplitude of low-frequency fluctuation; R, right; L, left; IOG, Inferior occipital gyrus; PG, Precentral gyrus.

Further, the ROC curve demonstrated that the ALFF differences in those regional brain areas alone discriminated the acute SD status from the RW status with high degrees of sensitivities (82.16 ± 7.61%; 75–93.8%) and specificities (81.23 ± 11.39%; 62.5–93.7%) with cut-off points of −0.351, 0.206, 0.2065, 0.1155, −0.8015, −0.405, and −0.3095 (mean beta signal value), respectively (Table 2, Figure 3).

**Table 2:** ROC curve for the ALFF differences in brain areas between SD and RW.

**Brain area**	**AUC**	**Sensitivity (%)**	**Specificity (%)**	Cut off point^*^
R_Cerebellum Anterior Lobe	0.805	75	81.2	−0.351
L_ Inferior Occipital Gyrus	0.875	75	87.5	0.206
R_Inferior Occipital Gyrus	0.809	81.3	75	0.2065
L_Thalamus	0.867	87.5	75	0.1155
L_Insula	0.82	93.8	62.5	−0.8015
L_ Postcentral Gyrus	0.848	75	93.7	−0.405
R_ Postcentral Gyrus	0.93	87.5	93.7	−0.3095

* *Cut off point of mean ALFF signal value*.

*ROC, Receiver operating characteristic; ALFF, Amplitude of low-frequency fluctuation; SD, Sleep deprivation; RW, Rested wakefulness; AUC, Area under the curve; R, Right; L, Left*.

### Pearson correlation analysis

**Figure 4:**
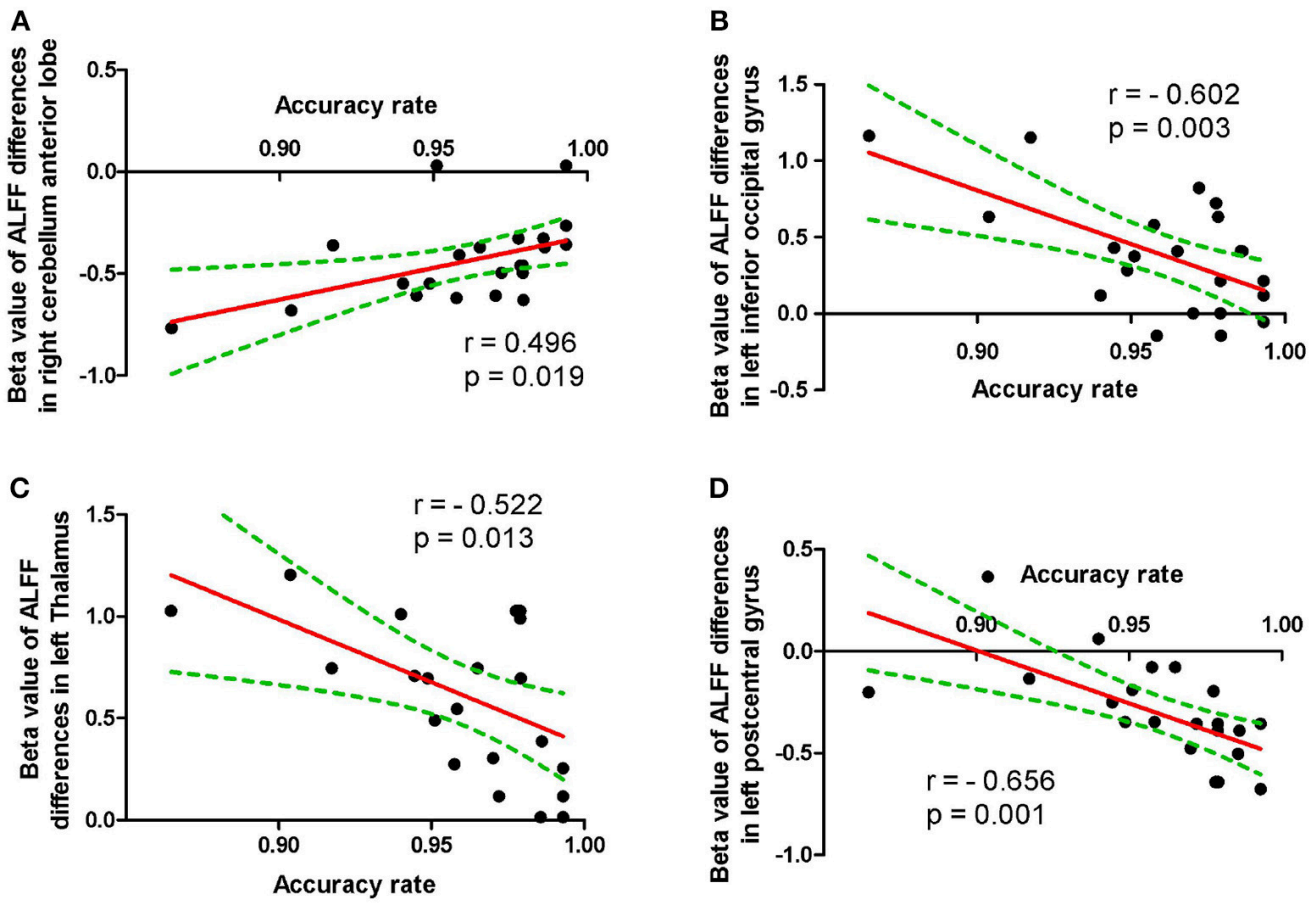
Pearson correlation between behavioral performances and beta values of ALFF differences in brain areas. Pearson correlation between accuracy rate and cerebullum **(A)**, left inferior occipital gyrus **(B)**, left thalamus **(C)** and left postcentral gyrus **(D)**. ALFF, Amplitude of low-frequency fluctuation.

The accuracy rate demonstrated a positive correlation with the ALFF value in the right cerebellum anterior lobe (*r* = 0.496, *p* = 0.019; Figure 4A), and negative correlations with the ALFF values in the left inferior occipital gyrus (*r* = −0.602, *p* = 0.003; Figure 4B), left thalamus (*r* = −0.522, *p* = 0.013; Figure 4C) and left postcentral gyrus (*r* = 0.656, *p* = 0.001; Figure 4D) during the acute SD status, respectively. None of the other correlations between the ALFF values in those different areas and the behavioral performances during the acute SD status were found (*p* > 0.05).

## Discussion

In the present study, we used ALFF analysis to demonstrate the differences in regional brain areas associated with acute SD, and their correlations with the clinical performances. Specifically, we found that SD was associated with widespread regional brain activities with lower ALFF values in the right cerebellum anterior lobe, and with higher ALFF values in the bilateral inferior occipital gyrus (BA 18, 19), left thalamus, left insula (BA 13), and bilateral postcentral gyrus. Furthermore, during the SD status, the accuracy rate showed correlations with the beta value of ALFF differences in those brain areas. Recently, the ROC curve is widely used to evaluate the reliability of a neuroimaging technique in distinguishing one group from another group (3, 22, 24). In general, the AUC value is considered as excellent between 0.9 and 1, considered as good between 0.8 and 0.9, considered as fair between 0.7 and 0.8, considered as poor between 0.6 and 0.7, and considered as failed between 0.5 and 0.6. In the present study, the ROC curve revealed that the ALFF differences in those brain areas had good discriminating abilities with a high AUC value (> 0.8). Further diagnostic analysis showed that these areas discriminated the SD status from the RW status with high degrees of sensitivities (mean, 82.16 ± 7.61%; 75–93.8%) and specificities (mean, 81.23 ± 11.39%; 62.5–93.7%).

In a previous study, a total of 16 healthy subjects (8 females, 8 males) were recruited, and SD was found to be associated with several ALFF differences in brain areas (29); however, the study did not take the gender differences into account. Previous studies have shown that there are wide gender differences in brain activity in healthy subjects both at the SD status and the RW status, and in patients with chronic insomnia relative to good sleepers in sleep neuroimaging studies (6, 24). In this framework, the present study only recruited healthy female subjects to exclude the effect of the gender factor. Specifically, we found that SD was associated with widespread regional brain activities with lower ALFF values and higher ALFF values, and this finding is different from that of the previous study. Therefore, our findings support the standpoint that the gender factor should be taken into account in the neuroimaging studies of sleep disorders (6, 24).

The hyperarousal and increased glucose utilization in patients with chronic primary insomnia were found in neurocognitive, neuroimaging, and physiological studies (30–32). Hyperarousal refers to magniloquent cognitive, somatic, and/or cortical activation, further leading to increased sensory information processing (33, 34), which is a core predisposing factor of chronic primary insomnia (35). Previous neuroimaging studies also found hyperarousal reactivation in several brain areas in individuals after SD and patients with chronic primary insomnia (6, 24). The present study found that SD is associated with increased ALFF areas in widespread regional brain areas, and these increased ALFF areas show negative correlations with the accuracy rate. There are two prevalent speculations for the increased regional brain activities (36). One explanation of the hyperarousal model could be that this is a brain compensation mechanism. Previous diffusion tensor imaging study showed that 23 h SD is associated with widespread fractional anisotropy decreases in several brain areas and as the waking prolonged the decreases become larger (37). Another explanation of the increased ALFF areas in widespread regional brain areas may be that the hyperactivation in these widespread regional brain areas may be interpreted as an enhanced neural effort to offset these decreased brain structures associated with SD. A previous task study found that the parietal lobe was not activated after normal sleep but was activated after SD (38). The occipital lobe and postcentral gyrus were found with higher regional homogeneity and ALFF values (6, 17); the thalamus was also found with higher ALFF value after acute SD (17), and the thalamus and insula were activated by acupuncture after acute SD (39). These findings were consistent with our study, and may reflect dynamic, compensatory changes in cerebral activation after SD.

The lower ALFF values in brain areas may indicate a consistent decrease of regional neuronal activity with poor synchronization and without in order (6). Poor regulation of behaviors and emotions are core features of SD. The cerebellum is involved in coordinating movement, and emotional and cognitive functions (6, 24), and associated with the aberrant regional brain activity in sleep disorders, such as patients with primary insomnia (24, 40) and obstructive sleep apnoea (41). In the present study, SD compared with RW had a significant lower ALFF value in the right cerebellum anterior lobe, and the mean ALFF value in this area had a positive correlation with accuracy rate (*r* = 0.496, *p* = 0.019). The decreased regional brain activity in the right cerebellum anterior lobe may reflect that the sleep-deprived brain needs to attempt to recruit more specific brain areas with advanced cognitive functions to accomplish the cognitive performance because of a continuing decline in the cerebellum activity. Interestingly, Wang et al. showed different findings of altered SD-related regional brain activities in several areas (29). Since the gender factor may influence the results in the neuroimaging studies of sleep disorders (6, 24), we therefore speculated that the differences between our study and Wang et al.'s study may be associated with the gender factor.

## Conclusions

In summary, the ALFF analysis is a useful index to locate the underlying altered regional brain activities in individuals during the SD status relative to the RW status with high degrees of sensitivities and specificities. SD is associated with the model of excitation–inhibition imbalance of cortical activations. These findings expand our knowledge and may help in deeper understanding of the neurobiological mechanisms underlying acute SD. Furthermore, the gender factor should be taken into account in the neuroimaging studies of sleep disorders. However, there are several potential limitations that should be noted. First, our study has a relatively small sample size and future studies on a larger number of sample sizes are necessary to corroborate our findings. Second, in our study the design of replication is not addressed. Third, the electronystagmogram has been used to dynamically monitor the sleep.

## Author contributions

LC wrote the main manuscript text. JZ conceived and designed the whole experiment. LC and XQ collected the data. JZ analyzed the data.

### Conflict of interest statement

The authors declare that the research was conducted in the absence of any commercial or financial relationships that could be construed as a potential conflict of interest.
